# Digital health activism of patients with chronic diseases: discursive strategies and themes: Victoria Dudina

**DOI:** 10.1093/eurpub/ckac131.186

**Published:** 2022-10-25

**Authors:** V Dudina

**Affiliations:** Sociology, St. Petersburg State University, St. Petersburg, Russia

## Abstract

**Background:**

On the Internet, there are many online communities where people living with chronic life-threatening diseases can discuss their problems. On the one hand, digital health activism promotes patient empowerment and could lead to better disease self-management. But, on the other hand, digital health activism raises concerns as it undermines the monopoly of expert medical knowledge. The objective of the research was to reveal the main discursive strategies and themes represented in Russian-language online communities for people with chronic life-threatening diseases. It could help to gain a better understanding of the purposes and effects of digital health activism on patients.

**Methods:**

Data were collected through the popular Russian-language social networking site “Vkontakte”. We selected open groups with memberships of more than 1000 users devoted to HIV/AIDS, cancer, tuberculosis, and diabetes. Discourse analysis and thematic analysis were implemented.

**Results:**

Revealed discursive strategies include the patients’ rights advocacy and resistance to the established order of health care. Three main types of discursive themes were identified:

a. Overcoming shortcomings of the health care system such as lack of qualified doctors, medicine shortage, and unethical behavior of health care personnel.

b. Overcoming the monopolization of expert medical knowledge and criticizing some treatment approaches.

c. Overcoming stigma and discrimination associated with disease both in healthcare settings and in everyday life.

**Conclusions:**

Research on digital activism of patients with chronic life-threatening diseases makes it possible to identify hidden discrimination practices and patient rights violations in healthcare settings. Members of online patient communities turn from objects of care into informed active subjects who critically evaluate medical prescriptions, are active in finding the better treatment, and in defending their rights.

**Key messages:**

• The research contributes to the revision of the paternalistic model of patient-doctor interaction.

• Attention to digital activism is important for the identification of hidden issues in healthcare.The research was supported by RSF (project No 22-18-00261).

